# Epidemiology of systemic sclerosis in the Asia-Pacific region: a systematic review and meta-analysis

**DOI:** 10.1080/07853890.2025.2479238

**Published:** 2025-03-21

**Authors:** Ajanee Mahakkanukrauh, Chetta Ngamjarus, Porjai Pattanittum, Siraphop Suwannaroj, Patnarin Pongkulkiat, Tippawan Onchan, Chingching Foocharoen

**Affiliations:** ^a^ Department of Medicine, Faculty of Medicine, Khon Kaen University, Khon Kaen, Thailand; ^b^ Department of Epidemiology and Biostatistics, Faculty of Public Health, Khon Kaen University, Khon Kaen, Thailand

**Keywords:** Systemic sclerosis, scleroderma, scleroderma and related disorders, epidemiology, prevalence, incidence

## Abstract

**Background:**

The epidemiological profile of systemic sclerosis (SSc) in the population in Asia-Pacific countries might help in planning for improved future care and research direction.

**Objectives:**

We aimed to estimate the pooled incidence and pooled prevalence of systemic sclerosis (SSc) in Asia-Pacific countries.

**Methods:**

We conducted and reported the systematic review following the Preferred Reporting Items for Systematic Reviews and Meta-Analysis (PRISMA) statement of 2020. Databases searched include PubMed, SCOPUS, CINAHL, and ProQuest, and hand searching, with a focus on publications from 1 January 2000 to 31 July 2023.

**Results:**

A total of 456 records were identified from the searches, 10 articles were included for review: six reported the incidence of SSc; nine reported the prevalence of SSc. We noted considerable heterogeneity. Subgroup analyses categorized by the period of study before and after the launch of the 2013 ACR/EULAR Classification Criteria for SSc demonstrated that both incidence and prevalence of SSc were significantly different between subgroups. The incidence of SSc before and after the launch was 1.85 per 100,000 (4 studies, I^2^ = 100%, 95%CI 0.53–6.40) and 9.61 per 100,000 (2 studies, I^2^ = 100%, 95%CI 4.90–18.85), respectively. The prevalence of SSc before and after the launch was 6.47 per 100,000 (6 studies, I^2^ = 97%, 95%CI 5.09–8.21) and 18.48 per 100,000 (3 studies, I^2^ = 100%, 95%CI 7.19–47.50), respectively.

**Conclusion:**

The epidemiology of SSc varied widely across the Asia-Pacific region depending on the study methodology and study period. The incidence of SSc in the Asia-Pacific region was estimated to be higher after the launch of the new classification criteria.

## KEY MESSAGES

The incidence and prevalence of systemic sclerosis (SSc) vary widely across the Asia-Pacific region, influenced by differences in study methodologies.

The incidence of SSc in the Asia-Pacific has risen following the implementation of the classification criteria.

## Introduction

Systemic sclerosis (SSc) is a rare connective tissue disease that classically leads to skin thickness and internal organ fibrosis. The disease primarily affects the skin but can also involve internal organs, including the lungs, heart, gastrointestinal tract, and kidneys, leading to significant morbidity and mortality [1]. The exact etiology of SSc remains unknown. SSc is typically divided into two primary subsets: diffuse cutaneous SSc (dcSSc) and limited cutaneous SSc (lcSSc) [2]. In lcSSc, skin thickening is confined to the face and areas distal to the elbows and knees, while dcSSc is characterized by more widespread skin thickening [2] and it is linked to a poorer prognosis compared to lcSSc [3,4]. The existing reports show the average age of onset for SSc is between 40 and 60 years [5–8], with the ratio of women to men ranging from 1.5 to 17:1 [5,9].

Originally, the diagnosis of SSc was based on the diagnostic criteria of the American College of Rheumatology (ACR) from 1980 [10]. However, the diagnostic criteria could only screen patients who already exhibited clear symptoms of SSc. Additionally, it had low sensitivity and specificity for diagnosis [11]. As a result, the criteria were further revised in 2006 [12] and most recently in 2013 [11]. The ACR and the European League Against Rheumatism (EULAR) established the Classification Criteria for SSc to allow for quicker diagnosis and more practical application, with sensitivity and specificity reaching 91% and 92%, respectively [11]. The 2013 ACR/EULAR Classification Criteria are widely adopted for diagnosis, considering factors such as skin thickening, Raynaud’s phenomenon, nailfold capillaroscopy abnormalities, and the presence of autoantibodies specific to the disease [11].

Epidemiological studies are essential for understanding the impact of SSc, its geographic variations, and trends in the prevalence and incidence of the disease. Identifying the target population and the specific characteristics of SSc across different regions can help refine classification systems and optimize disease management strategies. Furthermore, epidemiological studies comparing past and present data are crucial for updating the epidemiological profile of SSc, particularly in the Asia-Pacific region, where the clinical features of SSc closely resemble those seen globally. Understanding the distribution of SSc in Asia-Pacific countries is essential, as it will help address regional healthcare needs and guide future research. Our study aims to estimate the incidence and prevalence of SSc in Asia-Pacific countries through a systematic review, contributing to a better understanding of the disease in this region and informing future care and research initiatives.

## Methods

We did and reported the systematic review among adult SSc patients following the Preferred Reporting Items for Systematic Reviews and Meta-Analysis (PRISMA) statement of 2020 [13]. The inclusion criteria were; a) SSc patients who were age greater than 18 years and was diagnosed as SSc according to either 1980 ACR or 2013 ACR/EULAR Classification Criteria for SSc or ICD-10 of systemic sclerosis (M34); b) having details of prevalence (either point prevalence or period prevalence) and/or incidence of SSc during 1 January 2000 to 31 July 2023; c) clearly defined population sources: outpatient clinic-based, hospital-based, and population databased; and, d) reporting from Asia-Pacific countries. We included all cohort and cross-sectional studies and excluded case reports, case series, qualitative studies, randomized and non-randomized controlled trials, systematic reviews, and review articles.

### Information resources

CN conducted a comprehensive search of four databases, including PubMed, SCOPUS, CINAHL, and ProQuest for articles published in English from 2000 until 31 July 2023. The full search strategies are shown in Supplementary file 1. We also conducted a hand searching of the reference list from relevant articles.

### Study selection

All records from research results were retrieved and removed the duplicates in the Mendeley reference manager [https://www.mendeley.com/reference-management/reference-manager]. Two authors (CF and AM) independently screened the titles and the abstracts and excluded the articles if the articles did not met our inclusion criteria. Full articles of the remaining relevant studies were evaluated for eligibility. Any disagreement was solved by consensus or discussed with the third authors (SS). The PRISMA flow diagram was used for visually summarization of the study selection process and the PRISMA checklist is available in Supplementary file 2.

### Data extraction and analysis

Two authors (CF and AM) independently extracted the data. The third authors (SS) were consulted to solve the discrepancies. The information extracted from each article included authors, country, publication year, denominator size, age, gender, person-years, age range, case definition, and the number of incident and prevalent cases, and diagnostic/classification criteria of SSc. The incidence and prevalence of SSc from the included studies were reported with their 95% confidence intervals (CIs) in forest plots.

CN and PP spot-checked the numerical data before entering it into the meta-analysis. We carried out a random-effects meta-analysis to produce the overall estimate for the incidence and prevalence of SSc with their 95% CIs. The visual inspection of the forest plots, Cochrane Q statistics, and I^2^ statistic were used to determine the heterogeneity (variation across studies). Substantial heterogeneity was identified if the p-value of the Cochrane Q statistic is less than 0.10 and I^2^ > 50%. When substantial heterogeneity was detected, subgroup analysis by diagnostic criteria (before and after the launch of the 2013 ACR/EULAR Classification Criteria for SSc) was performed. Chi-squared test was used to examine the differences between subgroups. For robustness of results, we planned to conduct a sensitivity analysis by excluding survey study (using interview) from the analysis. However, we did not perform the sensitivity analysis because the survey study found a few cases with the lowest weight in the meta-analysis. A funnel plot was not created for evaluating a publication bias because there were fewer than 10 studies in the meta-analysis. The findings from meta-analysis were presented as forest plots and the data were analysed using R with the ‘meta’ package [14,15].

### Study quality assessment

Two authors (CF and AM) was independently assess the risk of bias of each included studies using the Strengthening the reporting of observational studies in epidemiology (STROBE) statement [16].

## Results

**Figure 1.**
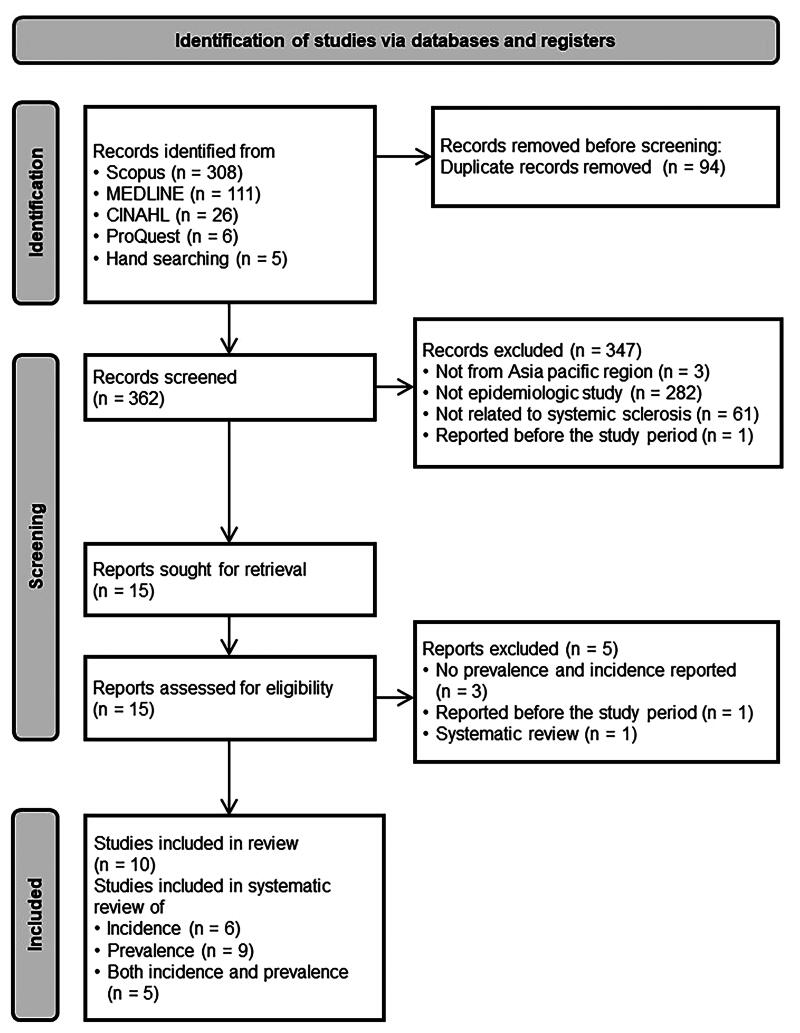
The PRISMA 2020 flow diagram.

The search identified 456 records, of which 94 duplicates were removed, leaving 362 unique records for title and abstract screening. Fifteen records were moved on to the full-text screening. Five were excluded due to the absence of reported incidence or prevalence of SSc (three studies), reporting before the study period (one study), and being a systematic review (one study). Consequently, 10 articles were included for review, with nine reporting on the prevalence of SSc and six also reporting on the incidence (Figure 1).

## Incidence of SSc in Asia-Pacific region

Six articles were included in the systematic review to define the incidence of SSc according to the inclusion criteria of our study method. Three articles were conducted in Taiwan, one in each country as follows: Japan, South Korea, Thailand (Table 1).

**Table 1.** Summary of characteristics of studies reporting of SSc incidence in Asia Pacific region.

Study	Kuo et al. [12]	See et al. [13]	Yu et al. [17]	Kang et al.	Kuwana et al. [14]	Foocharoen et al. [15]
Study design	Cross-sectional study	Cross-sectional study	Cross-sectional study	Cross-sectional study	Cross-sectional study	Cross-sectional study
Setting/context	Taiwan National Health insurance database	Taiwan National Health insurance database	Taiwan National Health insurance database	Korean Rare Intractable Disease registry database	Japanese Medical Data Center (JMDC) and the Medical Data Vision (MDV)	Ministry of Public Health database
Criteria diagnosis for SSc	NA (a review based on clinical data review by rheumatologists)	NA (a review based on clinical data review by rheumatologists)	NA (a review based on clinical data review by rheumatologists)	Criteria predefined by the government (identical to ACR criteria)	NA	NA
Source of information (such as medical record-ICD10, nationwide database-ICD10)	ICD-9	ICD-9	ICD-9	ICD-10	ICD-10	ICD-10
City and country	Taiwan	Taiwan	Taiwan	South Korea	Japan	Thailand
Years / time frame of data collection	2002–2007	2005	2000–2008	2008–2013	1 September 2016 to 31 August 2019	2020
Participant Characteristics						
Inclusion	Whole population	Whole population	Whole population with 1,000,0000 population sampling	Whole population	Whole population	Whole population
Exclusion	None	None	None	None	≤ 12 months of continued enrolment prior to index date	None
Number of case	1,479	54	118	2,402	933 from JMDC database 4,805 from MDV database	4,459
Number of total population	135,688,073 person-years	4,954,934	963,355	300,250,000 person-years	6,881,421	65,421,139
Result of incidence; per 100,000 person-years (95%CI)						
Overall	1.09	1.09 (0.82–1.42)	12.25 (10.14–14.67)	0.80	13.56 (12.70–14.46) (JMDC) (age > 20 years)	6.82 (6.62–7.02)
Female	1.74	1.6 (1.1–2.1)	2.5 (2.1–3.0)	NA	NA	8.7 (8.4–9.0)
Male	0.47	0.6 (0.3–0.9)	0.4 (0.2–0.6)	NA	NA	4.8 (4.6–5.1)
Age (years); mean ± SD	51.3 ± 15.2	NA	NA	NA	NA	58.2 ± 13.2
Age group	20–29 years: 0.38 30–39 years: 0.71 40–49 years: 1.76 50–59 years: 2.72 60–69 years: 3.05 70–79 years: 2.57 ≥ 80 years: 1.32	NA (peak age in female 40–49 years and male ≥ 70 years)	NA	NA	NA	≤29 years: 0.6 (0.5–0.7) 30–39 years: 2.2 (2.0–2.6) 40–49 years: 5.9 (5.4–6.4) 50–59 years: 13.6 (12.9–14.4) 60–69 years: 20.9 (19.8–22.0) ≥70 years: 15.8 (14.8–17.0)
dcSSc subset	NA	NA	NA	NA	NA	NA
lcSSc subset	NA	NA	NA	NA	NA	NA

ACR; American College of Rheumatology, SD; standard deviation, 95%CI; 95% confidence interval, JMDC; Japanese Medical Data Center, MDV; the Medical Data Vision, NA; no data available, dcSSc; diffuse cutaneous systemic sclerosis, lcSSc; limited cutaneous systemic sclerosis.

A cross-sectional study using the Taiwan National Health Insurance database from 2002 to 2007 by Kuo et al. [18] extracted information using ICD-9 codes. The authors reported an incidence of SSc of 1.09 per 100,000 person-years from a total of 135,688,073 person-years. The incidence was higher in females than in males (1.74 vs. 0.47 per 100,000 person-years). The mean age was 51.3 years, with the peak incidence occurring between the ages of 60 and 69 years.

The second cross-sectional study from Taiwan was conducted by See et al. [19] who used the Taiwan National Health Insurance database in 2005, overlapping with the period of the previous study. Of a total population of 4,954,934, fifty-four SSc new cases of SSc were identified using ICD-9 codes resulted in an incidence rate of 1.09 per 100,000 person-years. The incidence for females was 1.6 per 100,000 person-years with the peak age of incidence occurring at 40–49 years, whereas the incidence for males was 0.6 per 100,000 person-years and the peak age of incidence was ≥ 70 years. However, no SSc subsets were reported.

The third cross-sectional study from Taiwan, reported by Yu et al. used the same database as the two previously mentioned studies but sampled 1,000,000 population. The period of study was from 2000 to 2008. After excluding non-surviving cases and missing data, 963,355 sample population were evaluated to determine the incidence of SSc. The respective overall incidence of SSc, and incidence of SSc in females, and males, was reported as 1.5, 2.5, and 0.4 per 100,000 person-years. However, no ages of the SSc cases were included.

Kang et al. [17] reported the incidence of SSc in the South Korean population using the Korean Rare Intractable Disease registry database, covering the entire population from 2008 to 2013. The authors conducted a cross-sectional study using the diagnostic criteria predefined by the government, which did not differ from the ACR criteria. Out of 300,250,000 person-year, the incidence of SSc was 0.8 per 100,000 person-years. However, the incidence categorized by sex, SSc subsets and age group was not reported.

A study reported by Kuwana et al. [20] conducted an observational study using two databases in Japan (Japanese Medical Data Center; JMDC and the Medical Data Vision; MDV). Between 1 September 2016, and 31 August 2019, 933 individuals from the JMDC database and 4,805 from the MDV database were identified as having a new diagnosis of SSc based on ICD-10 codes. The authors reported the incidence of SSc using JMDC of 6.6 per 100,000 person-years. The respective incidence of SSc among females and males was 11.8 and 2.6 per 100,000 person-years. Among the entire population of 6,881,421, the 933 individuals with age > 20 years from the JMDC database were identified having SSc diagnosed by ICD-10, resulted in a prevalence of SSc 13.56 per 100,000 person-years. However, the age group for the peak incidence and the incidence categorized by SSc subset were not reported.

Foocharoen et al. [21] reported the incidence of SSc among Thais using Ministry of Public Health database from 2017 to 2020, diagnosing SSc based on ICD-10 codes. The authors revealed 4,701 new SSc cases out of 65,406,320 population in 2018, 4,983 new SSc cases out of 65,557,054 in 2019, and 4,459 new SSc cases out of 54,421,139 in 2020, resulting in respective incidences of 7.2, 7.6, and 6.8 per 100,000 person-years. The incidence of SSc among females in 2018, 2019, and 2020 was 9.4, 9.8, and 8.7 per 100,000 person-years, respectively, while the incidence of SSc among males was 4.9, 5.3, and 4.8 per 100,000 person-years, respectively. The mean age was comparable from 2018 to 2020 (59.7, 58.9, and 58.2 years, respectively). The highest incidence of the disease was observed in the Northeastern region from 2018 to 2020, with the respective incidences of 11.6, 12.1, and 11.1 per 100,000 person-years. However, there was no incidence of SSc categorized by SSc subset.

### The estimates of SSc incidence of in Asia Pacific region

**Figure 2.**
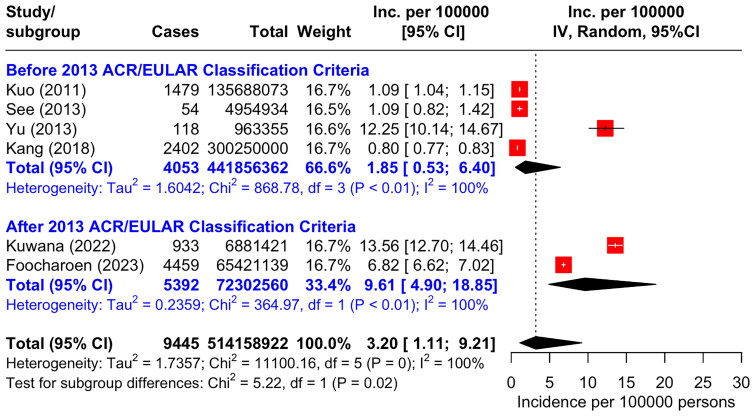
Forest plot of SSc incidence per 100,000 person-years categorized by the launch of the 2013 ACR/EULAR classification criteria of systemic sclerosis.

The pooled incidence by random-effects model after excluding missing data and death cases was 3.20 per 100,000 person-years (95%CI 1.11 to 9.21) with an I^2^ value of 100% (Figure 2). We analysed subgroup categorized by the period of study before and after launching the 2013 ACR/EULAR Classification Criteria for SSc, four studies were included in the group of before the classification criteria was launched and two were in the group of after the launch with the respective pooled incidence before and after the launch of 1.85 per 100,000 (95%CI 0.53 to 6.40) and 9.61 per 100,000 (95%CI 4.90 to 18.85) (Figure 2). However, considerable heterogeneities were found in both subgroups.

## Prevalence of SSc in Asia Pacific region

Nine full-texts were reviewed for determining the prevalence of SSc in Asia-Pacific region. There were four studies conducted in Taiwan, two studies in Japan, one in each country as follows: China, South Korea, Thailand (Table 2).

**Table 2.** Summary of characteristics of studies reporting of SSc prevalence in Asia Pacific region.

Study	Kuo et al. [12]	Li et al. [19]	See et al. [13]	Ohta et al. [20]	Yu et al. [18]	Kuo et al. [17]	Kim et al. [21]	Kuwana et al. [15]		Foocharoen et al. [16]
Study design	Cross-sectional study	Observational survey study	Cross-sectional study	Cross-sectional study	Cross-sectional study	Cross-sectional study	Cross-sectional study	Cross-sectional study		Cross-sectional study
Setting/context	Taiwan National Health insurance database	Community survey 10 out of 42 villages from Beijing	Taiwan National Health insurance database	Japanese nationwided database	Taiwan National Health insurance database	Taiwan National Health insurance database	Korean National Health Insurance	JMDC and MDV		Ministry of Public Health database
Criteria diagnosis for SSc	NA (a review based on clinical data review by rheumatologists)	ACR 1980	NA (a review based on clinical data review by rheumatologists)	NA	NA (a review based on clinical data review by rheumatologists)	NA	NA	NA		NA
Source of information (such as medical record-ICD10, nationwide database-ICD10)	ICD-9	Two steps survey Questionnaire Physical examination	ICD-9	NA	ICD-9	ICD-9	ICD-10	ICD-10		ILD-10
City and country	Taiwan	Beijing, China	Taiwan	Japan	Taiwan	Taiwan	South Korea	Japan		Thailand
Years / time frame of data collection	2002–2007	NA	2005	2007	1996–2008	2010	2012–2016	1 September 2016 to 31 August 2019		2017
Participant Characteristics										
Inclusion	Whole population	Permanent living in China	Whole population with 1,000,0000 population sampling	Whole population	Whole population with 1,000,0000 population sampling	Whole population from 1 Jan 1996 to 31 Dec 2010	Whole population	Whole population		Whole population
Exclusion	None	Denied 2^nd^ step evaluation	None	None	None	None	None	≤12 months of continued enrolment prior to index date		None
Number of case	1,284	1	70	10,058	37	1,891	2,648 (in 2012) 3,606 (in 2016)	2,459 from JMDC database (age > 20 years)		15,920
Number of total population	22,800,000 in 2007	10,556	1,000,000	126,000,000	963,355	23,658,577 in 2010	49,855,796 (in 2016)	6,881,421 (age > 20 years)		65,204,797
Result of prevalence (95%CI)										
Overall (per 100,000 population)	5.63 (mean prevalence from 2002–2007)	9.47 (0.24–52.77)	7.00 (5.46–8.84)	7.98 (7.83–8.14)	3.8 (2.7–5.3)	7.99	5.3 (in 2012) 7.2 (in 2016)	35.73 (34.34–37.17) (JMDC)		24.42 (24.04–24.80)
Female (per 100,000 population)	NA	1 out of 10,556	10.5 (7.7–13.3)	7.1 (6.9–7.2)	6.4 (1.7–9.1)	13.0	NA	NA		32.7 (32.1–33.3)
Male (per 100,000 population)	NA	0	3.4 (1.8–5.1)	0.92 (0.87–0.97)	1.4 (0.4–2.9)	3.0	NA	NA		15.8 (15.4–16.2)
Age (years); mean ± SD	NA	NA	NA	Mean age at onset Female: 50.2 ± 13.7 Male: 54.0 ± 14.1	NA	NA	NA	JMDC database: 49.0 ± 13.5 DMV database: 67.2 ± 13.7		59.7 ± 13.3
Age group (per 100,000 population)	NA	NA	NA	Peak prevalence 65–69 years	NA	NA	Peak prevalence 60–69 years	JMDC database <20 year: 4.9 20–29 years: 3.9 30–39 years: 11.5 40–49 years: 24.2 50–59 years: 34.0 60–69 years: 19.2 70–79 years: 2.4 ≥80 years: 0	DMV database <20 year: 0.6 20–29 years: 1.0 30–39 years: 2.3 40–49 years: 7.5 50–59 years: 13.2 60–69 years: 25.7 70–79 years: 32.4 ≥80 years: 17.3	≤29 years: 1.5 (1.4–1.7) 30–39 years: 5.9 (5.4–6.4) 40–49 years: 15.8 (15.0–16.6) 50–59 years: 47.3 (85.9–90.7) 60–69 years: 88.2 (85.9–90.7) ≥70 years: 86.9 (84.1–89.7)
dcSSc subset (per 100,000 population)	NA	NA	NA	NA	NA	NA	NA	NA		NA
lcSSc subset (per 100,000 population)	NA	NA	NA	NA	NA	NA	NA	NA		NA

ACR; American College of Rheumatology, SD; standard deviation, 95%CI; 95% confidence interval, JMDC; Japanese Medical Data Center, MDV; the Medical Data Vision, NA; no data available, dcSSc; diffuse cutaneous systemic sclerosis, lcSSc; limited cutaneous systemic sclerosis.

Kuo et al. [18] conducted a cross-sectional study using the Taiwan National Health Insurance database from 2002 to 2007, which covered at least 99% of entire population. The information was extracted using ICD-9, and the clinical and laboratory data were comprehensively assessed and requires a review based on clinical data review by rheumatologists. The mean prevalence from 2002 to 2007 was 5.63 per 100,000 populations.

Kuo et al. [22] also conducted the study with using the same database as the previous study but included the database in different period. The study included the whole population from Taiwan from 1 January 1996 to 31 December 2010. There were 1,891 cases with SSc out of total population of 23,658,577 in 2010, so the prevalence of SSc in 2010 was 7.99 per 100,000 population. The respective prevalence of the disease in females and males was 13.0 and 3.0 per 100,000 population. No report of mean age at diagnosis from both studies.

Li et al. [23] collected data through a community-based survey of eight common rheumatic diseases, including SSc from 10 out of 42 villages in Beijing, China, using a two-stages survey. The first stage was a questionnaire screening, and the second stage comprised a physical examination of those who had a positive response. The diagnosis of rheumatic diseases was based on the ACR criteria. Individuals who denied the second stage evaluation were excluded. Only 1 case (female) from the total population of 10,566 was identified as having SSc, resulting in a prevalence of 9.47 per 100,000 population. No data were reported regarding the time frame of data collection and age group.

See et al. [19] conducted a cross-sectional study using the Tiawan National Health Insurance database, with a randomly sampled population of 1,000,000 in 2005. SSc cases were identified using ICD-9 codes. Of those in the sampled population, 70 individuals were identified as having SSc with a prevalence of 7.00 per 100,000 population (95%C 5.46–8.84). The prevalence of female was 3 times more than male (10.5 VS. 3.4 per 100,000 population). However, no criteria for diagnosis and mean age were reported.

A study reported by Ohta et al. [24] conducted using Japanese nationwide database. Of the entire population of 126,000,000 in 2007, 10,058 were diagnosed with SSc. However, no specific criteria for diagnosis and source of information for SSc extraction were identified. The prevalence of SSc in Japan in 2007 was 7.98 per 100,000 population. The respective prevalence of SSc among females and males was 7.1 and 0.92 per 100,000 population, with the ratio of 7.7:1. The peak prevalence occurred in the 65–69 years age group, with the mean age at onset being 50.2 years in female and 54.0 years in males.

A cross-sectional study of Yu et al. [25] using data from the Taiwan National Health Insurance database from 1996 to 2008 with 1,000,000 population sampling and extracted using ICD-9. After excluding non-surviving cases and missing data, 963,355 sample population were evaluated to determine the prevalence of SSc. The prevalence of SSc was 3.84 per 100,000 population and the respective prevalence in females and males was 6.4 and 1.4 per 100,000 population with female to male ratio of 4.5:1. The mean age at onset was not mentioned in this study.

Kim et al. [26] conducted a cross-sectional study using Korean National Health Insurance database in 2012–2016 and extracted data using ICD-10. The authors reported that the prevalence of SSc in 2012 was 5.3 per 100,000 population, whereas in 2016 was increased to 7.23 per 100,000 population. The peak prevalence was between 60 and 69 years.

A cross-sectional study using two databases in Japan, the JMDC (a database of a health insurance societies-based claims) and the MDV (a database of a hospital claims), was reported by Kuwana et al. [20] The data collection period was from 1 September 2016 to 31 August 2019. Individuals with less than 12 months of continuous enrollment after the data collection period were excluded from the study. Among the entire population of 6,881,421, the 2,459 individuals with age > 20 years from the JMDC database were identified having SSc diagnosed by ICD-10, resulting in a prevalence of SSc 35.73 per 100,000 population. The mean age was 51.5 years, and the prevalence was much higher among those aged ≥ 65 years (189.5 vs 33.5 per 100,000 population).

An epidemiologic study conducted Thailand using the Ministry of Public Health database from 2017 to 2020 and diagnosing SSc based on ICD-10, revealed 15,920 cases of SSc out of the entire population of 65,204,797 [21]. The crude prevalence of SSc among Thai was 24.4 per 100,000 population, with the mean age of 59.7 years, and peak of the prevalence was within 60–69 age group. The prevalence of the disease was 32.7 and 15.8 per 100,000 population in female and male Thais, respectively. The authors did not specify the diagnostic criteria for SSc.

None of the full articles included in the systemic review reported the prevalence of SSc categorized by SSc subset.

### The estimates of SSc prevalence of in Asia Pacific region

**Figure 3.**
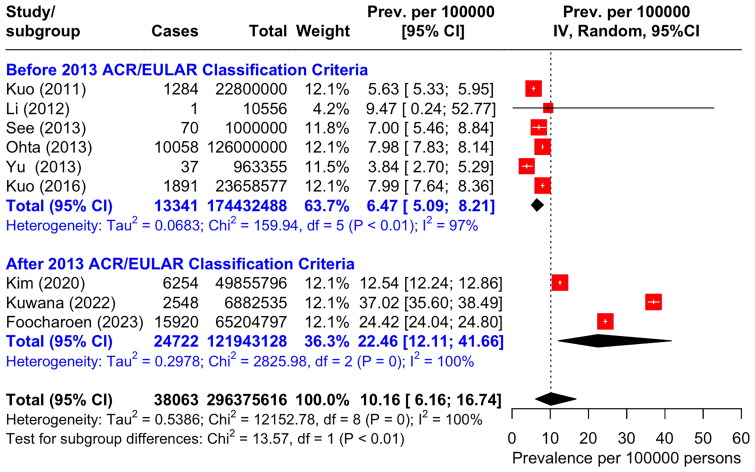
Forest plot of SSc prevalence per 100,000 population with subgroup analysis categorized by before and after the launch of 2013 ACR/EULAR classification criteria for SSc.

Given the substantial heterogeneity of the studies (9 studies, I^2^ = 100%), a pooled prevalence estimated by random-effects model after excluding missing data and death cases was 9.46 per 100,000 population (95%CI 5.76 to 15.55) (Figure 3).

We analysed subgroup categorized by the period of study before and after launching the 2013 ACR/EULAR Classification Criteria for SSc, six studies were included in the group of before the classification criteria was launched, and three were in the group of after the launch with the respective pooled prevalence before and after the launch of 6.47 per 100,000 (95%CI 5.09 to 8.21) and 18.48 per 100,000 (95%CI 7.19 to 47.50) (Figure 3).

We initially planned to conduct a sensitivity analysis to assess the impact of a survey-based study using interviews (Li et al.). However, since only a single case of SSc was diagnosed, the analysis would not have provided meaningful insights. As a result, we decided not to proceed with the sensitivity analysis.

Each study is characterized by various parameters, including, setting/context, criteria diagnosis for SSc, source of information, city, country, years/data collection, participant characteristics, exclusion criteria, number of cases, number of total populations, and results of prevalence (Table 2).

**Figure 4.**
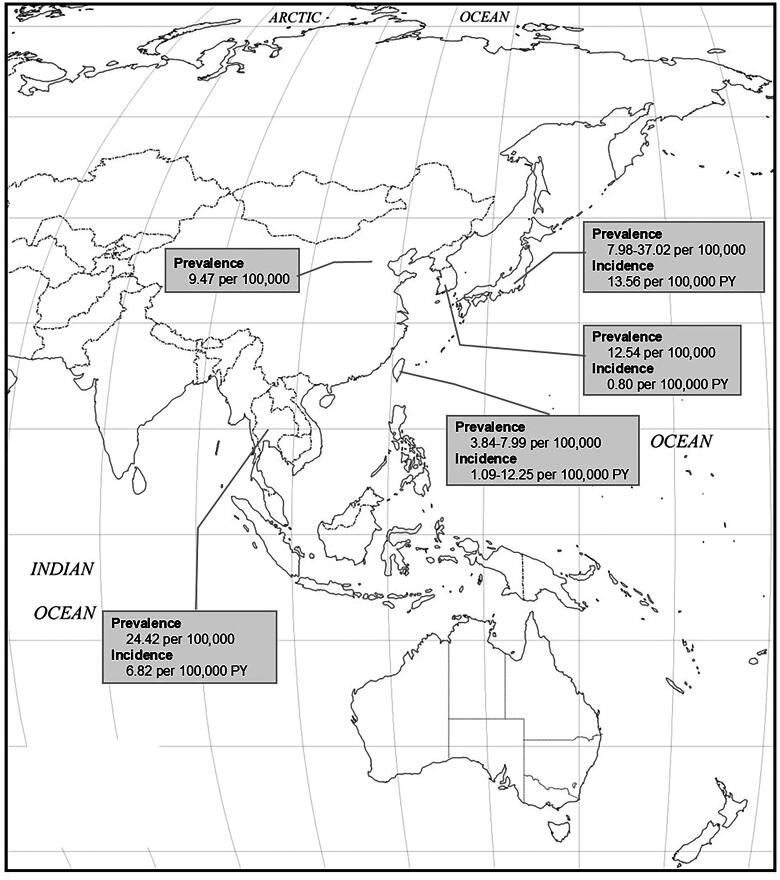
SSc Epidemiological data in the Asia-Pacific region (map from https://amaps.com/mapstoprint/OUTLINE%20MAPS/free_map_of_asia_oceania.htm).

The reviewed epidemiological data of SSc in the Asia-Pacific region, based on our methods, are presented as a map (Figure 4). The map highlights the limitations of the geographical representativeness of SSc epidemiology in this region.

## Risk of bias in included studies

**Figure 5.**
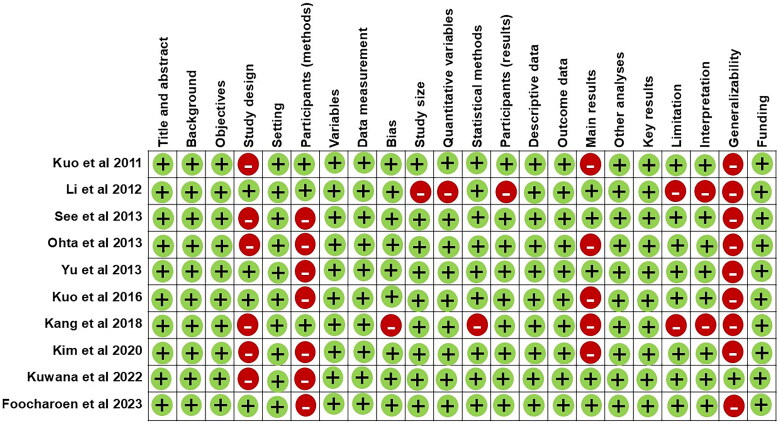
Quality of studies assessment by STROBE statement.

The risk of bias assessment tool for prevalence studies revealed that almost all studies had a relevant target population using national databases, except for one study that conducted sampling *via* community survey (Li et al. [23]). Two studies lacked a source of information for determining the case definition of SSc. Five out of ten studies evaluated diagnoses confirmed by physical examination (Li et al. [23]), by a review based on clinical data review by rheumatologists (Kuo et al. [18], See et al. [19], and Yu et al. [25]), or by a predefined criteria by the government (Kang et al. [17]) while the remaining studies relied on either ICD-9 or ICD-10 as a source of information, which may affect the accuracy of diagnosis. The quality of the study assessment using STROBE statement is presented in Figure 5.

## Discussion

We conducted a systematic review of the epidemiology of SSc in Asia-Pacific countries. This study included prevalence and incidence data covering literature in English between 2000 and 31 July 2023. The epidemiologic studies of SSc in the Asia-Pacific region were mostly from East Asia, with one from Southeast Asia. No reports were found from West and Central Asia. The included studies might not represent the data from the whole Asia-Pacific region because we did not include non-English literature in the study; thus, epidemiologic study conducted in non-English languages from those areas would not be reported.

Our study period of interest was from 2000 to 2023. According to the 2013 ACR/EULAR Classification Criteria for SSc, the sensitivity for diagnosing SSc, particularly early SSc, was higher than that of the 1980 ACR criteria [11]. We aimed to explore whether the incidence and prevalence of SSc in the Asia-Pacific region changed after the launch of the 2013 ACR/EULAR Classification Criteria for SSc. Therefore, we included data from 10 years before and 10 years after the classification criteria were implemented in daily practice. However, only 10 studies met our inclusion and exclusion criteria for full article review, of which nine were included in the analysis for prevalence and six for incidence of SSc.

In our review, there were four studies conducted in Taiwan which used the same database (Taiwan National Health Insurance database) and were conducted before the launch of the classification criteria. The period of data collection overlapped between the studies (2002–2007 in Kuo et al. [18], 2005 in See et al. [19], and 1996–2008 in Yu et al. [25]), so the incidence and prevalence reported in these studies might be identical. In contrast, the data collection periods for the two studies from Japan (2007 in Ohta et al. [24] and 2016–2019 in Kuwana et al. [20]) did not overlap. Although there was a 1-year overlap in the data collection periods of the two studies from South Korea (2008–2012 in Kang et al. [17] and 2013–2016 in Kim et al. [26]), these studies focused on incidence and prevalence, respectively. Therefore, the epidemiologic data from both Japan and South Korea may represent changes in incidence and/or prevalence over different periods.

The incidence and prevalence of SSc in Asia-Pacific region vary across different studies, with the respective rates ranging from 0.8 to 13.6 per 100,000 person-years and 3.8–35.7 per 100,000 population. The incidence before and after the launch of the classification criteria was 0.8–12.3 versus 6.8–13.6 per 100,000 person-years. According to the methodology of the enrolled study, the majority used ICD codes to identify SSc from the database. We assume that the ICD codes in the database may have been applied using the 2013 ACR/EULAR Classification Criteria for diagnosing SSc. The findings supported the better sensitivity of the classification criteria for identifying SSc patients. The prevalence and incidence of SSc were much higher in the study that included data collection after the 2013 ACR/EULAR Classification Criteria for SSc was launched. The prevalence of SSc before the launch of the classification criteria ranged from 3.8 to 9.5 per 100,000 population, whereas it ranged from 7.2 to 35.7 per 100,000 population after the launch as well as the incidence of SSc.

Although a subgroup analysis by the launch of the new classification criteria for SSc was evaluated, heterogeneity was still detected in the prevalence studies both before and after the launch of the classification criteria. This heterogeneity might be explained by the different methods used in the studies, included in the group before the launch of the new classification criteria and the overlapping time in the group after the launch. In the group before the launch, one study (Li et al. [23]) detected only one SSc case through a community survey, while the remaining studies used database to investigate the prevalence of the disease. In contrast, one study (Kim et al. [26]) in the group after the launch used a database that included periods both before and after the launch (2012 and 2016, respectively). Therefore, the interpretation of the prevalence of SSc from this analysis should be approached with caution.

The previous study found the various incidence and prevalence of SSc around the world [27]. The data from 39 studies of incidence and 61 studies of prevalence from the review showed that the respective global pool incidence rate and prevalence of SSc was 1.4 per 100,000 person-years (95%CI 1.1 to1.9) and 17.6 per 100,000 (95%CI 15.1 to 20.5). The North America had higher estimates of SSc than other regions and women were 5 times higher in both pooled incidence and pooled prevalence than men [27]. However, the study included the incidence and prevalence before using the 2013 ACR/EULAR Classification Criteria for SSc [11] which is more sensitive and more specificity [11] for diagnosis of SSc than the previous 1980 ACR criteria of SSc [10], LeRoy and Medsger 2011 [28], and the preliminary criteria for the very early diagnosis of SSc in 2011 [12]. There has been no study on the incidence and prevalence of SSc since the 2013 ACR/EULAR Classification Criteria for SSc.

Tian et al. also reported variations in the global incidence and prevalence of SSc in 2023 [29]. The study included both adult and juvenile SSc cases, with the respective incidence and prevalence for adults with SSc being 12.9 per 100,000 person-years and 27.9 per 100,000 population. Both incidence and prevalence rates were higher than those in our study. However, this review primarily included epidemiological data from high-income countries in Europe, North America, and the Asia-Pacific region, while data from Southeast Asia, South Asia, and other low-income regions—where limited investigative resources may contribute to delayed SSc diagnosis—were lacking. Additionally, most included studies applied a combined diagnostic approach using both the 1980 ACR criteria and the 2013 ACR/EULAR Classification Criteria for SSc. The authors also did not perform a subgroup analysis to compare epidemiological data before and after the introduction of the new classification criteria.

Following the launch of the 2013 ACR/EULAR Classification Criteria for SSc, the incidence of SSc in Asia-Pacific region was higher than that reported in a previous global systemic review by Bairkdar et al. [27], which included two studies from Europe and one study from South America (6.8 versus 2.1 per 100,000 person-years). The disparities might be related to the different methods of study. Bairkdar et al. [27] categorized incidence based on case definition methods of 1980 ACR criteria, LeRoy classification and subsets, LeRoy and Medsger Classification Criteria for Early Systemic Sclerosis in 2001, the 2013 ACR/EULAR Classification Criteria for SSc, ICD codes, and other/doctors opinion [27]. While all studies included in our systematic review used ICD codes for defining SSc cases. In general, ICD-10 coding errors can occur in daily practice, potentially leading to misdiagnosis within the database. However, a study by De Almeida Chaves et al. evaluated the reliability of ICD-10 in discharging SSc patients reported an overall positive predictive value of 93%, indicating the reliability of ICD-10 codes for SSc diagnoses in hospital databases. Although the use of ICD codes for case definition may result in either overestimation or underestimation of SSc incidence rate, the overall accuracy may not significantly impact incidence rates, as indicated by the reliability of ICD-10 coding reported by De Almeida Chaves et al. Consequently, differences in SSc incidence rate between the Asia-Pacific region and other areas may not solely stem from variations in methods of study.

Another factor that might explain the disparities in the epidemiology of SSc between the Asia-Pacific region and other zones is ethnicity. The proportion of SSc subsets from the previous reports of Southeast Asia and East Asia countries were commonly diffuse cutaneous SSc (dcSSc) [5,30] or comparable between limited cutaneous SSc (lcSSc) and dcSSc [9,31,32]. Conversely, reports from Europe, the United States, Australia, and New Zealand showed a higher frequency of lcSSc [8,33–39]. Additionally, differences in genetic factors, particularly HLA gene, were observed between countries. HLA-DRB1 and HLA-DPB1 were common among Asian populations [40–45], whereas HLA-DQA1 and HLA-DQB1 were revealed among European and American populations [46]. These findings may explain the variations in SSc distribution and epidemiology among different populations.

When categorizing the epidemiology of SSc by gender, females had a higher prevalence and incidence compared to males, with female to male ratios ranging from 7.7:1 to 2:1. The Japanese population seemed to have a slightly higher female to male ratio compared to the Taiwanese and Thai populations. These findings were not unexpected; however, the female to male ratio were slightly lower than those reported outside the Asia-Pacific region (2–7:1 in this study versus 4–8:1 from outside Asia-Pacific regions) [8,35–39,47–49], regardless of the period since the launch of the 2013 ACR/EULAR Classification Criteria for SSc.

The peak prevalence is observed in various age groups, with differences between databases. The majority of cases were of an average age between 40 and 69 years. One of the nine study in our systematic review for the prevalence of SSc included the age at onset, which was 50.2 years for female and 54.0 years for male with SSc [24]. The age at onset in this study seemed to be higher than the previous report by Coral-Alvarado et al. [50] which performed a global analysis of SSc in 2009. The authors reported the respective average age at onset among Asian, European, North American, and Latin American populations as 34, 48, 52, and 51 years [50]. The high sensitivity for diagnosis early SSc with the 2013 ACR/EULAR Classification Criteria for SSc should lead to earlier detection of SSc cases, so the average age at onset should be lower than in the past. However, the findings are contradictory. This discrepancy might be explained by the era of an Aging Society, where the prevalence and incidence of SSc might be shifting to and older population [51]. Notable, the most recent data on the incidence of SSc by Foocharoen et al. showed that new cases of SSc were highest in the 60–69 age group. Nevertheless, the exact reason for these findings is uncertain. A global evaluation is suggested to assess and explain the shift in the age at onset of SSc.

A high degree of heterogeneity (I^2^ close to 100%) was observed in our analysis, indicating substantial variability among the included studies. This heterogeneity may be due to differences in study methodologies, including variations in case definitions, data collection methods, and study designs. To further investigate heterogeneity, we considered conducting subgroup analysis to assess the impact of potential moderators, such as diagnostic criteria and SSc subsets, on the epidemiology of SSc. However, since no studies reported epidemiological data categorized by SSc subsets, we were unable to determine whether this was a potential source of heterogeneity. e transition from earlier classification systems to the 2013 ACR/EULAR Classification Criteria, may have influenced the reported incidence and prevalence rates. Furthermore, variations in population characteristics, environmental factors, genetic predisposition, and healthcare accessibility across different geographic locations likely contributed to the observed differences in epidemiological estimates. Given this high heterogeneity, caution is warranted when interpreting the findings, as direct comparisons between studies may be challenging. Nevertheless, by identifying gaps in current knowledge, our findings emphasize the need for standardized methodologies and consistent diagnostic criteria in future epidemiological research on SSc.

Our study had several limitations. First, a small number of studies included during the period of interest, which may affect the robustness of the findings and impact the statistical power of meta-analysis, potentially leading to variability in effect estimates and wider confidence intervals. Second, all the studies included were from East and Southeast Asia, meaning the results may not fully represent the entire Asia-Pacific region. While our initial intention was to provide a broader perspective on the Asia-Pacific region, we recognize that the current dataset does not cover all subregions, as no epidemiological data were reported from countries outside East Asia (China, Japan, and Korea) and Southeast Asia (Thailand). Although these findings may not ­represent the entire Asia-Pacific region, this systematic review and meta-analysis provide valuable insights into an overlooked aspect of SSc epidemiology in the region and underscore the need for further investigation in other Asia-Pacific countries. Lastly, only studies published in English were reviewed, introducing the possibility of publication and language bias. Nevertheless, this systematic review sheds light on the epidemiology of SSc in the Asia-Pacific region. The results can guide the importance of expanding research efforts to underrepresented regions within the Asia-Pacific, ensuring a more comprehensive understanding of disease distribution. Future studies with larger, more representative samples and harmonized study designs are needed to refine epidemiological estimates and strengthen our understanding of SSc in the Asia-Pacific region.

## Conclusion

Both the incidence and prevalence of SSc varied widely across the Asia-Pacific region, depending on the study methodology. This variation can be attributed to differences in diagnostic criteria and population characteristics. Due to this observed heterogeneity and the limited number of studies providing data on the incidence and prevalence of SSc, caution is warranted when interpreting the findings. Standardized methodologies are needed to accurately assess the epidemiology of SSc. Future research should aim to include a broader range of populations within the Asia-Pacific region.

## Supplementary Material

Supplemental Material

## Data Availability

The original contributions presented in the study are included in the article. Further inquiries can be directed to the corresponding authors.

## Abbreviations

SSc: systemic sclerosis
PRISMA: Preferred Reporting Items for Systematic Reviews and Meta-Analysis
ACR: American College of Rheumatology
EULAR: European Alliance of Associations for Rheumatology
ICD-10: The International Classification of Diseases, Tenth Revision
ICD-9: The International Classification of Diseases, Ninth Revision
JMDC: Japanese Medical Data Centre
MDV: the Medical Data Vision
DcSSc: diffuse cutaneous systemic sclerosis
LcSSc: limited cutaneous systemic sclerosis
HLA: human leukocyte antigen
