# Supplementary material for: Epidemiology of systemic sclerosis in the Asia-Pacific region: a systematic review and meta-analysis
Source: Ann Med. 2025 Mar 21;57(1):2479238. doi: 10.1080/07853890.2025.2479238 (PMC11934167; doi:10.1080/07853890.2025.2479238)
Supplement: Supplemental Material [file IANN_A_2479238_SM8043.zip › supplementary_file/supplementary_file1.docx]

**PubMed search strategy on Aug 5, 2023**

| **Search number** | **Query** | **Results** |
| --- | --- | --- |
| 1 | "Scleroderma, Systemic"[Mesh]OR (systemic sclerosis[Title/Abstract] OR scleroderma[Title/Abstract]) | 32,885 |
| 2 | "Adult"[Mesh] NOT ("Child"[Mesh] OR "Infant"[Mesh]) | 7,071,402 |
| 3 | "Asia"[Mesh] AND "Pacific Ocean"[Mesh] | 789 |
| 4 | Afghanistan[Title/Abstract] OR Bahrain[Title/Abstract] OR Bangladesh[Title/Abstract] OR Bhutan[Title/Abstract] OR Brunei[Title/Abstract] OR Cambodia[Title/Abstract] OR China[Title/Abstract] OR Cyprus[Title/Abstract] OR Korea[Title/Abstract] OR Fiji[Title/Abstract] OR India[Title/Abstract] OR Indonesia[Title/Abstract] OR Iran[Title/Abstract] OR Iraq[Title/Abstract] OR Japan[Title/Abstract] OR Jordan[Title/Abstract] OR Kazakhstan[Title/Abstract] OR Kiribati[Title/Abstract] OR Kuwait[Title/Abstract] OR Kyrgyzstan[Title/Abstract] OR Lao[Title/Abstract] OR Lebanon[Title/Abstract] OR Malaysia[Title/Abstract] OR Maldives[Title/Abstract] OR Marshall Islands[Title/Abstract] OR Micronesia[Title/Abstract] OR Mongolia[Title/Abstract] OR Myanmar[Title/Abstract] OR Nauru[Title/Abstract] OR Nepal[Title/Abstract] OR Oman[Title/Abstract] OR Pakistan[Title/Abstract] OR Palau[Title/Abstract] OR Papua New Guinea[Title/Abstract] OR Philippines[Title/Abstract] OR Qatar[Title/Abstract] OR Korea[Title/Abstract] OR Samoa[Title/Abstract] OR Saudi Arabia[Title/Abstract] OR Singapore[Title/Abstract] OR Solomon Islands[Title/Abstract] OR Sri Lanka[Title/Abstract] OR Syrian Arab Republic[Title/Abstract] OR Tajikistan[Title/Abstract] OR Thailand[Title/Abstract] OR Timor-Leste[Title/Abstract] OR Tonga[Title/Abstract] OR Turkey[Title/Abstract] OR Turkmenistan[Title/Abstract] OR Tuvalu[Title/Abstract] OR United Arab Emirates[Title/Abstract] OR Uzbekistan[Title/Abstract] OR Vanuatu[Title/Abstract] OR Viet Nam[Title/Abstract] OR Yemen[Title/Abstract] OR Macao[Title/Abstract] OR Taiwan[Title/Abstract] OR Hong Kong[Title/Abstract] | 1,015,767 |
| 5 | "epidemiology" [Subheading] NOT ("Randomized Controlled Trials as Topic"[Mesh] OR "Randomized Controlled Trial" [Publication Type] OR "Qualitative Research"[Mesh] OR "Case Reports" [Publication Type]) | 2,519,326 |
| 6 | (#1 AND #2) AND (#3 OR #4) | 200 |
| 7 | (#5 AND #6) AND ("1990/01/01"[Date - Publication] : "2023/07/31"[Date - Publication]) | 111 |

**Scopus search strategy on Aug 5, 2023 (found 308 records)**

( ( ( ( TITLE-ABS-KEY ( systemic AND sclerosis ) OR TITLE-ABS-KEY ( systemic AND scleroderma ) ) ) AND ( ( TITLE-ABS-KEY ( adult* ) AND NOT TITLE-ABS-KEY ( child* ) AND NOT TITLE-ABS-KEY ( infant ) ) ) ) AND ( ( ( TITLE-ABS-KEY ( asia-pacific ) OR TITLE-ABS-KEY ( asia AND pacific ) ) ) OR ( TITLE-ABS-KEY ( afghanistan ) OR TITLE-ABS-KEY ( bahrain ) OR TITLE-ABS-KEY ( bangladesh ) OR TITLE-ABS-KEY ( bhutan ) OR TITLE-ABS-KEY ( brunei ) OR TITLE-ABS-KEY ( cambodia ) OR TITLE-ABS-KEY ( china ) OR TITLE-ABS-KEY ( cyprus ) OR TITLE-ABS-KEY ( korea ) OR TITLE-ABS-KEY ( fiji ) OR TITLE-ABS-KEY ( india ) OR TITLE-ABS-KEY ( indonesia ) OR TITLE-ABS-KEY ( iran ) OR TITLE-ABS-KEY ( iraq ) OR TITLE-ABS-KEY ( japan ) OR TITLE-ABS-KEY ( jordan ) OR TITLE-ABS-KEY ( kazakhstan ) OR TITLE-ABS-KEY ( kiribati ) OR TITLE-ABS-KEY ( kuwait ) OR TITLE-ABS-KEY ( kyrgyzstan ) OR TITLE-ABS-KEY ( lao ) OR TITLE-ABS-KEY ( lebanon ) OR TITLE-ABS-KEY ( malaysia ) OR TITLE-ABS-KEY ( maldives ) OR TITLE-ABS-KEY ( marshall AND islands ) OR TITLE-ABS-KEY ( micronesia ) OR TITLE-ABS-KEY ( mongolia ) OR TITLE-ABS-KEY ( myanmar ) OR TITLE-ABS-KEY ( nauru ) OR TITLE-ABS-KEY ( nepal ) OR TITLE-ABS-KEY ( oman ) OR TITLE-ABS-KEY ( pakistan ) OR TITLE-ABS-KEY ( palau ) OR TITLE-ABS-KEY ( papua AND new AND guinea ) OR TITLE-ABS-KEY ( philippines ) OR TITLE-ABS-KEY ( qatar ) OR TITLE-ABS-KEY ( korea ) OR TITLE-ABS-KEY ( samoa ) OR TITLE-ABS-KEY ( saudi AND arabia ) OR TITLE-ABS-KEY ( singapore ) OR TITLE-ABS-KEY ( solomon AND islands ) OR TITLE-ABS-KEY ( sri AND lanka ) OR TITLE-ABS-KEY ( syrian AND arab AND republic ) OR TITLE-ABS-KEY ( tajikistan ) OR TITLE-ABS-KEY ( thailand ) OR TITLE-ABS-KEY ( timor-leste ) OR TITLE-ABS-KEY ( tonga ) OR TITLE-ABS-KEY ( turkey ) OR TITLE-ABS-KEY ( turkmenistan ) OR TITLE-ABS-KEY ( tuvalu ) OR TITLE-ABS-KEY ( united AND arab AND emirates ) OR TITLE-ABS-KEY ( uzbekistan ) OR TITLE-ABS-KEY ( vanuatu ) OR TITLE-ABS-KEY ( viet AND nam ) OR TITLE-ABS-KEY ( yemen ) OR TITLE-ABS-KEY ( macao ) OR TITLE-ABS-KEY ( taiwan ) OR TITLE-ABS-KEY ( hong AND kong ) ) ) ) AND ( ( ( TITLE-ABS-KEY ( epidemiology ) OR TITLE-ABS-KEY ( incidence ) OR TITLE-ABS-KEY ( prevalence ) OR TITLE-ABS-KEY ( occurrence ) ) ) AND NOT ( ( TITLE-ABS-KEY ( randomized AND controlled AND trial ) OR TITLE-ABS-KEY ( randomised AND control AND trial ) OR TITLE-ABS-KEY ( randomized AND clinical AND trial ) OR TITLE-ABS-KEY ( randomized AND controlled AND study ) OR TITLE-ABS-KEY ( qualitative AND research ) OR TITLE-ABS-KEY ( qualitative AND study ) OR TITLE-ABS-KEY ( case AND report ) ) ) ) AND PUBYEAR > 1990

**CINAHL Complete search strategy on Aug 5, 2023 (found 802 records)**

| **Search number** | **Query** | **Results** |
| --- | --- | --- |
| S1 | TI ( systemic sclerosis or scleroderma ) OR AB ( systemic sclerosis or scleroderma ) OR MH ( systemic sclerosis or scleroderma ) | 6,526 |
| S2 | TI ( adults or adult or aged or elderly ) OR AB ( adults or adult or aged or elderly ) OR MH ( adults or adult or aged or elderly ) NOT TI ( children or childhood or child or kids ) NOT AB ( children or childhood or child or kids ) NOT MH ( children or childhood or child or kids ) NOT TI ( infants or baby or newborn or neonate ) NOT AB ( infants or baby or newborn or neonate ) NOT MH ( infants or baby or newborn or neonate ) | 1,941,724 |
| S3 | (TI asia-pacific region OR AB asia-pacific region OR MH asia-pacific region) OR AB(Afghanistan) OR AB(Bahrain) OR AB(Bangladesh) OR AB(Bhutan) OR AB(Brunei) OR AB(Cambodia) OR AB(China) OR AB(Cyprus) OR AB(Korea) OR AB(Fiji) OR AB(India) OR AB(Indonesia) OR AB(Iran) OR AB(Iraq) OR AB(Japan) OR AB(Jordan) OR AB(Kazakhstan) OR AB(Kiribati) OR AB(Kuwait) OR AB(Kyrgyzstan) OR AB(Lao) OR AB(Lebanon) OR AB(Malaysia) OR AB(Maldives) OR AB(Marshall Islands) OR AB(Micronesia) OR AB(Mongolia) OR AB(Myanmar) OR AB(Nauru) OR AB(Nepal) OR AB(Oman) OR AB(Pakistan) OR AB(Palau) OR AB(Papua New Guinea) OR AB(Philippines) OR AB(Qatar) OR AB(Korea) OR AB(Samoa) OR AB(Saudi Arabia) OR AB(Singapore) OR AB(Solomon Islands) OR AB(Sri Lanka) OR AB(Syrian Arab Republic) OR AB(Tajikistan) OR AB(Thailand) OR AB(Timor-Leste) OR AB(Tonga) OR AB(Turkey) OR AB(Turkmenistan) OR AB(Tuvalu) OR AB(United Arab Emirates) OR AB(Uzbekistan) OR AB(Vanuatu) OR AB(Viet Nam) OR AB(Yemen) OR AB(Macao) OR AB(Taiwan) OR AB(Hong Kong) OR TI(Afghanistan) OR TI(Bahrain) OR TI(Bangladesh) OR TI(Bhutan) OR TI(Brunei) OR TI(Cambodia) OR TI(China) OR TI(Cyprus) OR TI(Korea) OR TI(Fiji) OR TI(India) OR TI(Indonesia) OR TI(Iran) OR TI(Iraq) OR TI(Japan) OR TI(Jordan) OR TI(Kazakhstan) OR TI(Kiribati) OR TI(Kuwait) OR TI(Kyrgyzstan) OR TI(Lao) OR TI(Lebanon) OR TI(Malaysia) OR TI(Maldives) OR TI(Marshall Islands) OR TI(Micronesia) OR TI(Mongolia) OR TI(Myanmar) OR TI(Nauru) OR TI(Nepal) OR TI(Oman) OR TI(Pakistan) OR TI(Palau) OR TI(Papua New Guinea) OR TI(Philippines) OR TI(Qatar) OR TI(Korea) OR TI(Samoa) OR TI(Saudi Arabia) OR TI(Singapore) OR TI(Solomon Islands) OR TI(Sri Lanka) OR TI(Syrian Arab Republic) OR TI(Tajikistan) OR TI(Thailand) OR TI(Timor-Leste) OR TI(Tonga) OR TI(Turkey) OR TI(Turkmenistan) OR TI(Tuvalu) OR TI(United Arab Emirates) OR TI(Uzbekistan) OR TI(Vanuatu) OR TI(Viet Nam) OR TI(Yemen) OR TI(Macao) OR TI(Taiwan) OR TI(Hong Kong) | 256,861 |
| S4 | TI ( epidemiology or incidence or prevalence or occurrence ) OR AB ( epidemiology or incidence or prevalence or occurrence ) OR MH ( epidemiology or incidence or prevalence or occurrence ) | 564,287 |
| S5 | TI ( randomized controlled trials or rtc or randomised control trials or randomized clinical trial or randomized controlled study ) OR AB ( randomized controlled trials or rtc or randomised control trials or randomized clinical trial or randomized controlled study ) OR MH ( randomized controlled trials or rtc or randomised control trials or randomized clinical trial or randomized controlled study ) OR TI ( qualitative research or qualitative study ) OR AB ( qualitative research or qualitative study ) OR MH ( qualitative research or qualitative study ) OR TI case report OR AB case report OR case report | 595,317 |
| S6 | (S1 AND S2 AND S3) AND (S4 NOT S5)  Limiters - Published Date: 19900101-20230731  Expanders - Apply equivalent subjects  Search modes - Find all my search terms | 26 |

**ProQuest** **search strategy on Aug 5, 2023**

| **Search number** | **Query** | **Results** |
| --- | --- | --- |
| S1 | publication(systemic sclerosis) OR publication(scleroderma) OR abstract(systemic sclerosis) OR abstract(scleroderma)  Limited by:Document type:Article | 10,139 |
| S2 | publication(adults or adult or aged or elderly ) OR abstract(adults or adult or aged or elderly ) NOT publication(children or childhood or child or kids) NOT abstract(children or childhood or child or kids) NOT publication(infants or baby or newborn or neonate) NOT abstract(infants or baby or newborn or neonate)  Limited by:Document type:Article | 1,419,268 |
| S3 | publication(asia-pacific region) OR abstract(asia-pacific region)  Limited by:Document type:Article | 3,680 |
| S4 | publication(Afghanistan) OR publication(Bahrain) OR publication(Bangladesh) OR publication(Bhutan) OR publication(Brunei) OR publication(Cambodia) OR publication(China) OR publication(Cyprus) OR publication(Korea) OR publication(Fiji) OR publication(India) OR publication(Indonesia) OR publication(Iran) OR publication(Iraq) OR publication(Japan) OR publication(Jordan) OR publication(Kazakhstan) OR publication(Kiribati) OR publication(Kuwait) OR publication(Kyrgyzstan) OR publication(Lao) OR publication(Lebanon) OR publication(Malaysia) OR publication(Maldives) OR publication(Marshall Islands) OR publication(Micronesia) OR publication(Mongolia) OR publication(Myanmar) OR publication(Nauru) OR publication(Nepal) OR publication(Oman) OR publication(Pakistan) OR publication(Palau) OR publication(Papua New Guinea) OR publication(Philippines) OR publication(Qatar) OR publication(Korea) OR publication(Samoa) OR publication(Saudi Arabia) OR publication(Singapore) OR publication(Solomon Islands) OR publication(Sri Lanka) OR publication(Syrian Arab Republic) OR publication(Tajikistan) OR publication(Thailand) OR publication(Timor-Leste) OR publication(Tonga) OR publication(Turkey) OR publication(Turkmenistan) OR publication(Tuvalu) OR publication(United Arab Emirates) OR publication(Uzbekistan) OR publication(Vanuatu) OR publication(Viet Nam) OR publication(Yemen) OR publication(Macao) OR publication(Taiwan) OR publication(Hong Kong)  Limited by:Document type:Article | 197,111 |
| S5 | abstract(Afghanistan) OR abstract(Bahrain) OR abstract(Bangladesh) OR abstract(Bhutan) OR abstract(Brunei) OR abstract(Cambodia) OR abstract(China) OR abstract(Cyprus) OR abstract(Korea) OR abstract(Fiji) OR abstract(India) OR abstract(Indonesia) OR abstract(Iran) OR abstract(Iraq) OR abstract(Japan) OR abstract(Jordan) OR abstract(Kazakhstan) OR abstract(Kiribati) OR abstract(Kuwait) OR abstract(Kyrgyzstan) OR abstract(Lao) OR abstract(Lebanon) OR abstract(Malaysia) OR abstract(Maldives) OR abstract(Marshall Islands) OR abstract(Micronesia) OR abstract(Mongolia) OR abstract(Myanmar) OR abstract(Nauru) OR abstract(Nepal) OR abstract(Oman) OR abstract(Pakistan) OR abstract(Palau) OR abstract(Papua New Guinea) OR abstract(Philippines) OR abstract(Qatar) OR abstract(Korea) OR abstract(Samoa) OR abstract(Saudi Arabia) OR abstract(Singapore) OR abstract(Solomon Islands) OR abstract(Sri Lanka) OR abstract(Syrian Arab Republic) OR abstract(Tajikistan) OR abstract(Thailand) OR abstract(Timor-Leste) OR abstract(Tonga) OR abstract(Turkey) OR abstract(Turkmenistan) OR abstract(Tuvalu) OR abstract(United Arab Emirates) OR abstract(Uzbekistan) OR abstract(Vanuatu) OR abstract(Viet Nam) OR abstract(Yemen) OR abstract(Macao) OR abstract(Taiwan) OR abstract(Hong Kong)  Limited by:Document type:Article | 1,468,493 |
| S6 | ([S1] AND [S2]) AND ([S3] OR [S4] OR [S5]) | 21 |
| S7 | publication(epidemiology) OR abstract(epidemiology) OR publication(incidence) OR abstract(incidence) OR publication(prevalence) OR abstract(prevalence) OR publication(occurrence) OR abstract(occurrence)  Limited by:Document type:Article | 1,588,810 |
| S8 | publication("randomized controlled trial" OR "randomised control trial") OR abstract("randomized controlled trial" OR "randomised control trial") OR publication("randomized clinical trial " OR "randomized controlled study") OR abstract("randomized clinical trial " OR "randomized controlled study") OR publication("qualitative research" OR "qualitative study ") OR abstract("qualitative research" OR "qualitative study ") OR publication("case report") OR abstract("case report")  Limited by:Document type:Article | 160,131 |
| S13 | [S6] AND ([S7] NOT [S8]) AND YR(1990-2023) | 6 |
